# Dr. Fell’s Cure for Cancer

**Published:** 1857-08

**Authors:** 


*Dr. Fell's Cure for Cancer.*—Dr. Fell was some time since admitted into the Middlesex Hospital for the purpose of experimenting upon patients with Cancer, with a secret remedy, with which he has been in possession some 15 years. He was admitted into the Hospital, upon the agreement that he should make known to the Surgical Staff the nature of the remedies used by him, and that within six months, he should publish to the world his treatment. The Boston Medical and Surgical Journal says: "It is but justice to Dr. Fell to say that the *surgical staff* of the *Middlesex Hospital*, in a report upon the result of his treatment, made March 18th 1857, speak favorably of his method." "They say that Dr. Fell's treatment is safe and easy of application; that it is confined to the enucleation of the tumor merely."

Dr. Fell has published a treatise upon Cancer and its treatment, in which his secret remedies are given; the principle ingredient being, Sanguinaria Canadensis, which he supposes has a specific effect upon Cancers. Not only upon the tumor itself, but in eradicating the Constitutional diathesis. He combines with the Canadensis, chloride of zinc; One part of the former to two of the latter. The blood root is given at the same time internally in combination with the iodide of arsenic, Cicuta or Taraxicum.

